# Case report: acute myocardial infarction in the setting of acute transcatheter aortic valve thrombus

**DOI:** 10.3389/fcvm.2023.1164668

**Published:** 2023-06-20

**Authors:** Elsa Hebbo, Alessandro El Khoury, Dounia Iskandarani, Fadi Sawaya

**Affiliations:** ^1^ Department of Medicine, American University of Beirut, Beirut, Lebanon; ^2^ Cardiology Department, American University of Beirut Medical Center, Beirut, Lebanon

**Keywords:** interventional cardiology. acute myocardial infarction, acute coronary syndrome, transcatheter aortic valve replacement, valve thrombus, thromboembolism, anticoagulation

## Abstract

We describe a case of valve thrombosis and a subsequent thromboembolic event within only 10 days of transcatheter aortic valve implantation (TAVI). Postprocedural anticoagulants are not standard of care medications post-TAVI in patients without atrial fibrillation. Valve thrombosis is an indication to initiate anticoagulation to resolve and prevent further thrombus.

## 1. Introduction

Transcatheter aortic valve implantation (TAVI) is used as an alternative for surgical aortic valve replacement in high-risk patients with severe aortic stenosis (AS). Despite continuous enhancement in the technique, periprocedural complications of TAVI remain of concern (1, 2). This is increasing in importance with its recent approval for usage in younger and healthier populations. There is almost a 10% risk of either thromboembolic or major bleeding events within 30 days of the procedure (1). Many factors such as the patient's prothrombotic state, procedural technique, and periprocedural medications can impact the occurrence of these complications. Hence, efforts are being made to understand their pathophysiology in order to adjust management accordingly.

**Figure 1:**
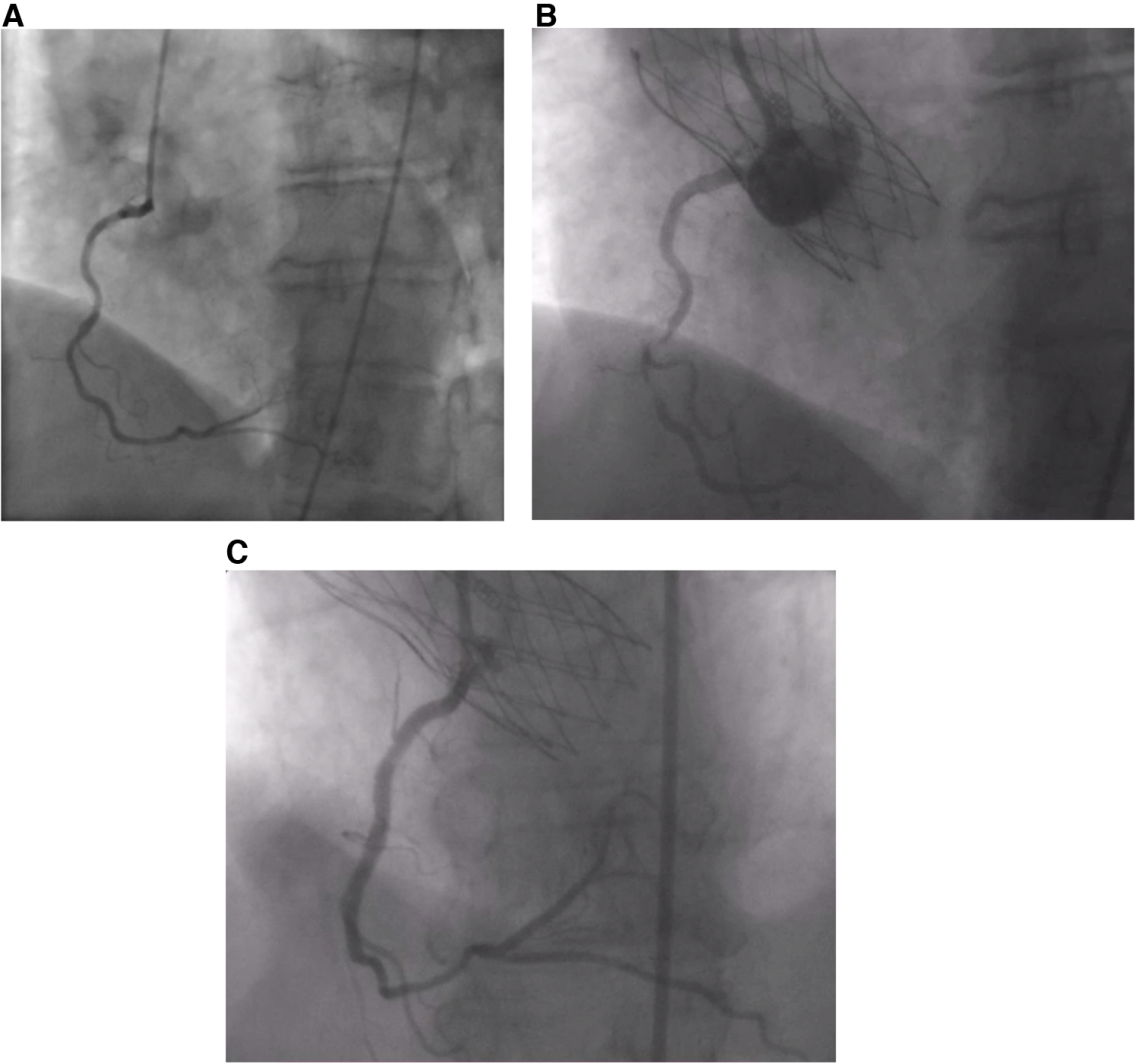
(**A**) Absence of CAD prior to the procedure. (**B**) Thrombus in RCA post-TAVI. (**C**) RCA post-stenting.

In this report, we describe a case of valve thrombosis and a subsequent thromboembolic event within only 10 days of the procedure. The patient presented with acute severe chest pain post-TAVI and was found to have ST-elevations in the inferior electrocardiogram (ECG) leads (Figure 1A).

## 2. Manuscript formatting

### 2.1. Case description

A 68-year-old female patient was referred to our institution for AS. She has a past medical history of hypertension and hypothyroidism as well as a recent diagnosis of urothelial carcinoma. She is a chronic smoker with a 30-pack-year history. Routine transthoracic echocardiography (TTE) reported a left ventricular ejection fraction of 60%–64% and a calcified aortic valve with a mean gradient (MG) of 40 mmHg and an aortic valve area of 0.8 cm^2^. Virtual basal ring sizing showed a perimeter of 70.2 mm^2^ with an area of 378.4 mm^2^ and a sinutubular junction of 24.6 mm. Pre-procedural cardiac catheterization showed non-obstructive coronary artery disease (Figure 1A). Further assessment with computed tomographic (CT) angiography demonstrated atherosclerotic calcifications of the aortic root, thoracic aorta, and coronary arteries and an incidental kidney tumor. Consequently, the heart team opted for TAVI given her need to undergo an urgent nephrectomy for her urothelial carcinoma.

A 25 mm Navitor valve was successfully deployed under local anesthesia with procedural coronary angiography showing no evidence of obstructive CAD. She had a transient third-degree atrioventricular block that resolved on the table with a normal ECG when she arrived at the cardiac care unit next day. TTE showed an MG 18 mmHg and trivial paravalvular leak. The patient was discharged home one day post-TAVI with a single antiplatelet therapy (SAPT), 100 mg of aspirin daily.

**Figure 2:**
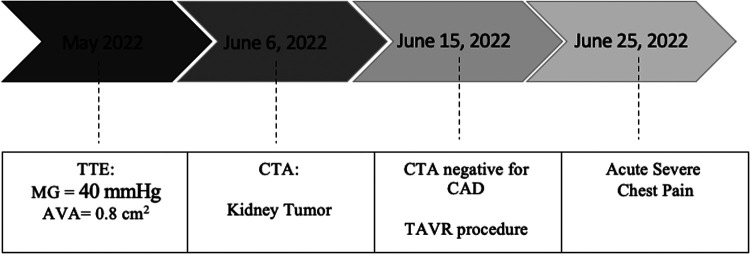
Timeline of imaging, symptoms, and procedures.

She was doing well until 10 days after the procedure when she presented with acute severe chest pain (Figure 2). Immediate ECG showed evidence of inferior myocardial infarction (MI) with ST elevations in leads II and III and ventricular fibrillation (aVF). High sensitivity troponin level was elevated reaching 50,000 pg/ml. She also had an elevated c-reactive protein and white blood count; however, she did not have any fever or positional chest pain. Her blood pressure was 120/60 mmHg. TTE showed evidence of inferior wall hypokinesis. Emergent coronary angiography revealed a thrombus occluding the second segment of the right coronary artery (RCA) (Figure 1B). Angioplasty was performed with implantation of one drug-eluting stent (Figure 1C). The patient's symptoms, ECG changes, and troponin elevations provided evidence in favor of MI instead of other differentials such as pericarditis or aortic dissection following the procedure. She was stabilized and discharged on 75 mg of clopidogrel, 100 mg of aspirin, 5 mg of amlodipine twice daily, and 2.5 mg of bisoprolol twice daily.

**Figure 3:**
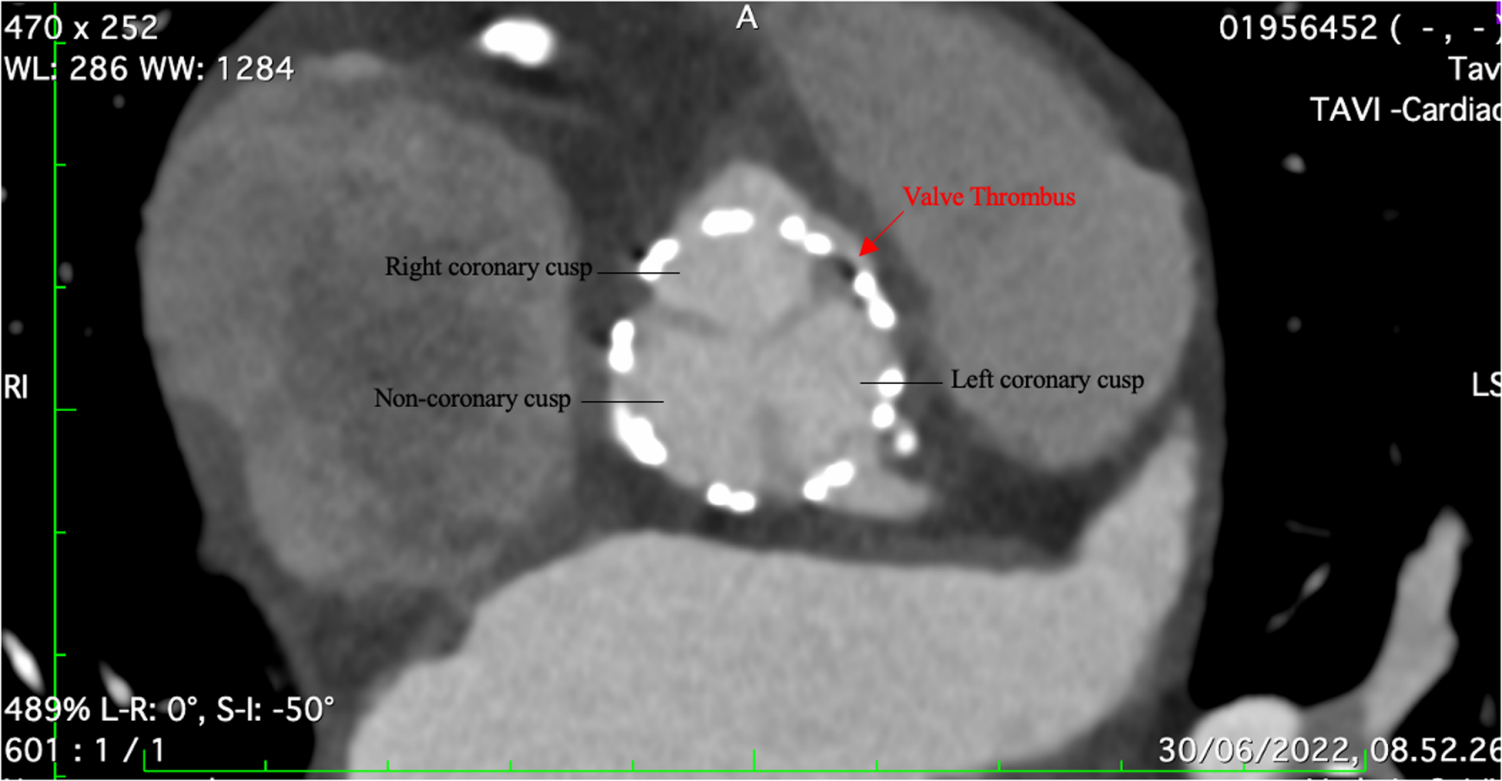
CT angiography showing a small thrombus between the right coronary and left coronary cusp.

The etiology of the MI was investigated with a follow-up CT angiography given that the patient did not have evidence of CAD before the procedure. The CT showed a small thrombus between the right coronary and left coronary cusp (Figure 3). The patient was then put on for two weeks on triple therapy: 100 mg of aspirin, 75 mg of clopidogrel, and 15 mg rivaroxaban. This was followed by clopidogrel and rivaroxaban for six weeks. There were no further cardiac or embolic clinical presentations, and the patient was cleared for her nephrectomy.

## 3. Discussion

Transcatheter valve thrombosis is defined by CT as a hypoattenuated leaflet thickening and by echocardiography as a subclinical mobile mass or a clinical dysfunction in the valve (1, 2). The dysfunction manifests as an increased transvalvular gradient, progressing regurgitation, or reduced valve diameter that usually responds to anticoagulation administration (2). Approximately 40% of patients with TAVI have asymptomatic valve thrombosis while clinical thrombosis occurs in up to 0.7% of patients per year. The most common complaint of valve thrombosis is dyspnea due to valve stenosis; however, thromboembolic events could occur leading to a wide range of presentations from stroke to MI (1, 3). The median duration from TAVI to thrombosis is 6 months (2, 3). Our case demonstrates an example of acute thrombosis, which is very rare. The underlying pathophysiology contributing to our patient's acute episode could be linked to her underlying malignancy and hypercoagulable state (3, 4). Other factors such as the type of valve, valve-in-vale procedures, increased valve size, only antiplatelet usage, and patient obesity are also associated with an increased risk of thrombosis (2, 4). Our patient was discharged as indicated by guidelines with SAPT only.

Thromboembolic events due to valve thrombosis mainly cause cerebrovascular accidents and transient ischemic strokes. The incidence of MI in the setting of valve thrombosis hasn't been mentioned in the literature except in a few case reports. Most cases report MI associated with valve thrombosis 4–15 months post-procedure; however, none mention an acute episode of both MI and valve thrombosis (5, 6). Another interesting case is that of a 79-year-old with typical features of non-ST elevation MI 7 months post-TAVI, but coronary angiography did not show evidence of occlusion. A cardiac CT scan revealed thrombosis of both the right and non-coronary leaflets of the valve. Cardiac MRI then showed evidence of transmural myocardial infarction (7).

To reduce the prevalence of valve thrombosis and its associated complications, efforts have been made to adjust management, especially in terms of postprocedural antithrombotic medications. According to the American Heart Association, Canadian Cardiovascular Society, and European Society of Cardiology, the standard current treatment post-TAVI is a single antiplatelet therapy (SAPT) (8–10). DAPT was considered the standard treatment of care until the management was challenged. Monotherapy with aspirin decreased all types of bleeding events and cardiovascular and all-cause mortality. However, valvular thrombosis was similar between both groups (11, 12). Oral anticoagulation (OAC) treatment prevents the formation of thrombus, but its usage is only limited to those with an indication such as atrial fibrillation and clinically significant valve thrombosis. The higher risk of bleeding with anticoagulation is a major concern (12). GALILEO and ATLANTIS studies in which rivaroxaban and apixaban were used, respectively, showed no clinical benefit to OAC. An increased risk of bleeding and even thromboembolism was noted with GALILEO (3, 12). As for patients with an indication for anticoagulation, monotherapy with OAC is indicated unless a recent percutaneous intervention was done. In this case, studies showed that the addition of P2Y12 inhibitor to OAC for up to 6 months was clinically effective (12). There are still no clear recommendations regarding the usage of OAC with subclinical valve thrombosis.

## Data Availability

The raw data supporting the conclusions of this article will be made available by the authors, without undue reservation.
